# Regulation of DNA Double Strand Breaks Processing: Focus on Barriers

**DOI:** 10.3389/fmolb.2019.00055

**Published:** 2019-07-16

**Authors:** Federica Marini, Chetan C. Rawal, Giordano Liberi, Achille Pellicioli

**Affiliations:** ^1^Dipartimento di Bioscienze, Università degli studi di Milano, Milan, Italy; ^2^Istituto di Genetica Molecolare Luigi Luca Cavalli-Sforza, CNR, Pavia, Italy; ^3^IFOM Foundation, Milan, Italy

**Keywords:** resection barriers, DSB processing, NHEJ, HDR, DNA:RNA hybrid

## Abstract

In all the eukaryotic cells, nucleolytic processing (resection) of a double strand DNA break (DSB) is a key step to channel the repair of the lesion toward the homologous recombination, at the expenses of the non-homologous end joining (NHEJ). The coordinated action of several nucleases and helicases generates 3′ single strand (ss) DNA, which is covered by RPA and recombination factors. Molecular details of the process have been first dissected in the model organism *Saccharomyces cerevisiae*. When DSB ends are occupied by KU, a central component of the NHEJ, the Mre11-Rad50-Xrs2 (MRX) nuclease complex (MRN in human), aided by the associated factors Sae2 (CTIP in human), initiates the resection process, inducing a nick close to the DSB ends. Then, starting from the nick, the nucleases Mre11, Exo1, Dna2, in cooperation with Sgs1 helicase (BLM in human), degrade DNA strand in both the directions, creating the 3′ ssDNA filament. Multiple levels of regulation of the break processing ensure faithful DSB repair, preventing chromosome rearrangements, and genome instability. Here we review the DSB resection process and its regulation in the context of chromatin. Particularly, we focus on proteins that limit DSB resection, acting as physical barriers toward nucleases and helicases. Moreover, we also take into consideration recent evidence regarding functional interplay between DSB repair and RNA molecules nearby the break site.

## DSB End Processing

DSBs are classically defined as broken chromosomes, however uncapped telomere ends and reversed forks are bound and processed by the same factors. In this review we generally focus on broken chromosomes, although proteins, and mechanisms that we mention are active on whole types of DSBs.

**Figure 1:**
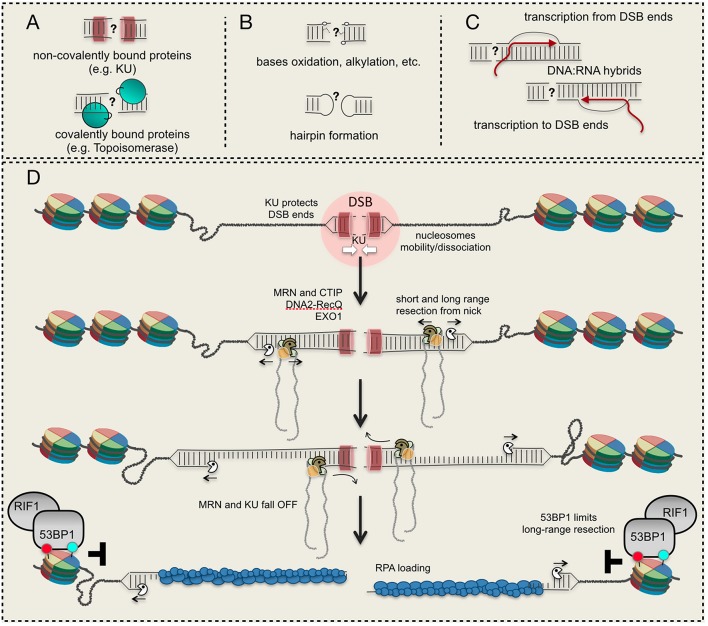
DSB ends and their processing. **(A)** Proteins bound to DSB ends interfere with resection initiation; **(B)** structural/chemical modifications of DNA ends require specific processing; **(C)** DNA:RNA hybrid formation at a DSB; **(D)** a model to resect a DSB starting from a nick induced by Mre11 nearby the DSB site. Red and light green circles indicate histone modifications. See details in the text.

In all the eukaryotes, a DSB can be repaired through non-homologous end joining (NHEJ) or homology directed recombination (HDR). Both pathways are organized in distinct steps and sub-pathways, which involve the coordination of several factors and enzymes (Heyer et al., 2010; Symington, 2016). Of note, specific mechanisms are required to process DSB ends containing covalently-bound proteins (such as Topoisomerase), DNA alterations (oxidation, methylation, hairpin formation, and others) and associated RNA molecules (e.g., DNA:RNA hybrids), which interfere with their repair through NHEJ and HDR (Figures 1A–C). Moreover, an irreparable DSB can be eventually processed by telomerase and DNA polymerase alpha-primase (Pol α-Prim), together with other factors, leading to *de novo* telomere addition (Putnam and Kolodner, 2017). Given the heterogeneity and complexity of the mechanisms involved, multiple levels of regulation have been identified, determining the repair of a DSB in the different cell cycle phases and chromatin context. Indeed, the uncontrolled DSB processing and repair greatly contribute to chromosome rearrangements (deletions, insertion, translocations), hallmarks of cancer and other pathological conditions associated to genome instability.

The nucleolytic processing (also called resection) of the DSB ends is a critical and finely regulated step to promote HDR over NHEJ. Indeed, the resection process generates an extended 3′-end ssDNA filament, which is then covered by RPA and the recombinase Rad51, depending on the sub-pathway (Symington, 2016).

The DSB resection is carried out by the coordinated actions of several nucleases, among which Mre11, Exo1, and Dna2 are the most involved from yeast to human. According to current models, Dna2 in cooperation with the helicase Sgs1 (BLM in human), and Exo1 process a DSB whose 5′ ends are accessible. Alternatively, a DSB with blocked or chemically modified ends needs the activity of the MRX (MRN in human) complex to initiate the process (Figure 1D). Indeed, *in vivo* and *in vitro* data (Neale et al., 2005; Shibata et al., 2014; Reginato et al., 2017; Wang et al., 2017, 2018) have shown that Mre11 is recruited nearby the DSB ends and induces a nick on the 5′-end filament, creating the entry point for both Exo1 and Dna2-Sgs1/BLM (Figure 1D). Then, starting from the nick, MRX/MRN processes the DNA in the 3′-to-5′ direction till the break site (short-range resection), while Exo1 and Dna2-Sgs1/BLM extensively process the DNA in the 5′-to-3′ direction (long-range resection) (Figure 1D). Interestingly, recent *in vitro* data indicate that BLM promotes the EXO1 resection processivity, too (Soniat et al., 2019). This nick-dependent mechanism for resection is activated in S and G2/M phases through the CDK1-dependent phosphorylation of Sae2 (CTIP in human) (Huertas et al., 2008; Huertas and Jackson, 2009), which associates with the Mre11 complex.

The importance of regulating the DNA ends resection in DSB repair is underlined by the increasing list of factors participating in the reaction in human cells, including the oncosuppressor BRCA1 (Zhao et al., 2019).

Below we review how specific factors and DNA/RNA transactions limit DSB resection, acting as physical barriers toward the nucleases. However, their antagonistic roles in the process appear very dynamic, likely exerting both negative and positive regulations on DSB repair.

## Nucleosome-Dependent Barrier

There is a general agreement that DNA, wrapped around the histone octamer into the nucleosome, is refractory to be resected due to steric hindrance. Indeed, in yeast the resection of DSBs frequently terminates at nucleosome (Mimitou et al., 2017); moreover, *in vitro* assays showed that DNA with reconstituted nucleosomes is resected by both Exo1 and Dna2-Sgs1 slower than naked DNA (Adkins et al., 2013). Remarkably, other *in vitro* results showed that BLM is able to slide nucleosomes, if RPA is added in the assay, promoting DNA resection by EXO1 and DNA2 (Xue et al., 2019). Of importance, the phosphorylation of RPA is critical to limit resection at nucleosomes, interfering with the strand-switching of BLM helicase (Soniat et al., 2019). However, Exo1 can actively resect DNA packed into nucleosomes containing the H2A.Z histone variant, which promotes higher mobility and instability of the octamer (Adkins et al., 2013). As such, the dynamic deposition of H2A.Z, together with other histone modifications, might facilitate the long-range resection by Exo1, with processing rate similar to naked DNA. On the other hand, it has been also shown that H2A.Z and H3.3 variants facilitate the loading of the NHEJ factors KU and XRCC4 onto DSB, thus limiting resection initiation (Xu et al., 2012; Luijsterburg et al., 2016). Nevertheless, other modifications of the histone core have been recently shown to facilitate the recruitment at DSB of both NHEJ and pro-resection factors, leading to a more complex scenario. According to several *in vivo* results, current models support a fundamental role of chromatin remodelers to mobilize and/or dissociate nucleosomes 1-2 kb nearby a DSB, creating the entry-space for repair factors (Shim et al., 2007; Price and D'Andrea, 2013;Clouaire and Legube, 2019; Figure 1D).

## KU-Dependent Barrier

Soon after a DSB formation, the heterodimer Ku70-80 complex (KU) binds DNA ends in all the cell cycle phases, acting as a platform for the association of several factors involved in NHEJ (Frit et al., 2019). Along with its role in promoting NHEJ, KU plays also fundamental role in limiting chromosome translocations mediated by the annealing of ssDNA repeats in human cells (Weinstock et al., 2006). Indeed, KU-bound DSB ends are resistant to Exo1 and Dna2 processing (Shim et al., 2010; Symington, 2016; Wang et al., 2018), reducing recombination DNA repair by micro-homology mediated end joining (MMEJ, also called alternative end-joining or alt-EJ in higher eukaryotes) and single strand annealing (SSA) mechanisms (Symington, 2016). In yeast, KU-dependent resection barrier is predominant in G1 phase (Clerici et al., 2008), when MRX-Sae2 is not activated by CDK1, or in the absence of functional MRX complex or Sae2 (Mimitou and Symington, 2010). Accordingly, deletion of *KU70* partially suppressed the resection defect and sensitivity of *sae2* or *mre11* mutants to ionizing radiations (Bonetti et al., 2010; Mimitou and Symington, 2010; Foster et al., 2011).

These and other experimental evidence support the involvement of the Mre11 complex and Sae2/CTIP to overcome the KU barrier, through the nick-dependent resection initiation (Figure 1D). By this model, the short-range resection through the Mre11 complex, together with Sae2/CTIP, is responsible for KU removal from the ends (Chanut et al., 2016; Symington, 2016), leading to a more complex and functional interplay between NHEJ and HDR. This mechanism is also functional at one-ended DSB created at broken DNA replication forks in human cells (Chanut et al., 2016). Moreover, depending upon the organisms, it is known that KU binding to DSB is finely regulated through neddylation (Brown et al., 2015), ubiquitylation (Postow et al., 2008; Feng and Chen, 2012), sumoylation (Hang et al., 2014), acetylation (Kim et al., 2014), and phosphorylation by DNA-PKs (Chan et al., 1999). In particular, neddylation primes ubiquitylation of KU in human cells, facilitating the release of the complex and associated factors from repaired DNA (Brown et al., 2015). Moreover, it has been shown that the AAA-ATPase p97 also cooperates for the removal of ubiquitinated KU from DSBs, after completion of end joining in human cells (van den Boom et al., 2016). However, it is unknown whether these and/or other post-translational modifications of KU might also control DSB resection initiation through KU stability at the DNA ends.

Further studies will be required to define how these multiple post-translational modifications of KU are conserved throughout evolution, co-exist during the cell cycle, regulate resection, and modulate DSB repair pathways.

## 53BP1-Dependent Barrier

The mammalian p53-binding protein 1 (53BP1) and its yeast ortholog Rad9 are important regulators of the DSB repair pathway choice (Panier and Boulton, 2014). Remarkably, 53BP1 and Rad9 act on all types of DSBs, including reversed forks and uncapped telomeres. They are recruited to chromatin through direct recognition of a DSB-specific histone code and their function as an anti-resection factor is conserved throughout evolution. Both 53BP1 and Rad9 act as mediators, linking the upstream kinase ATR/Mec1 to the downstream effector kinases CHK2/Rad53 and CHK1. In yeast Rad9 oligomers are recruited to chromatin through three different pathways: (1) the constitutive interaction with the histone H3 methylated at the K79 residue by Dot1/DOT1L; (2) the binding to the histone H2A phosphorylated at the S129 residue by Mec1; (3) the interaction with Dpb11/TOPBP1. All of these three pathways cooperate for efficient checkpoint signaling and cell survival after genotoxic treatments throughout the cell cycle.

In higher eukaryotes 53BP1 protects DNA ends from inappropriate 5′ hyper-resection, facilitating NHEJ, and error-free gene conversion at the expense of mutagenic SSA and alt-EJ (Ochs et al., 2016). Of note, extended ssDNA can lead to increased recombination events between repeats that are frequently present in eukaryotic genomes, leading to increased hypermutagenesis at breakpoint junctions (Sinha et al., 2017). Similarly to Rad9, 53BP1 recruitment requires the direct recognition of a DSB-specific histone code: it displays a strong binding affinity for the histone H4 constitutively mono- or di-methylated at the K20 (Botuyan et al., 2006) and for the histone H2A DSB-induced ubiquitination at K15 (Fradet-Turcotte et al., 2013). Moreover, 53BP1 oligomerization, mediated by DYNLL1, is essential for its recruitment to DSBs (Becker et al., 2018; West et al., 2019). Specifically, 53BP1 barrier is known to antagonize nucleases involved in the long-range resection, although its role to block resection initiation is supported by data in yeast, particularly in mutants affecting short-range resection. Interestingly, it has been shown that Rad9 accumulates at DSB ends in yeast cells lacking *SAE2*, blocking resection initiation by Dna2-Sgs1 (Bonetti et al., 2015; Ferrari et al., 2015; Yu et al., 2018). Moreover, resection initiation and capture of distant double-strand ends by CTIP is counteracted by 53BP1 in human cells (Guirouilh-Barbat et al., 2016).

**Figure 2:**
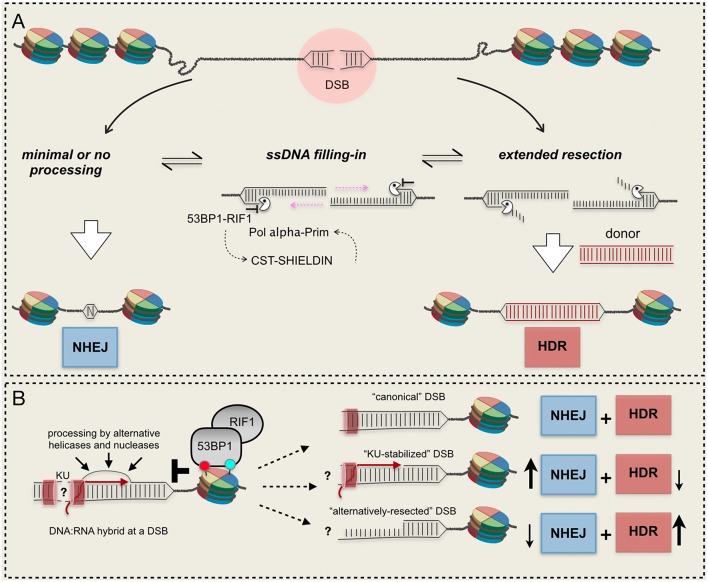
Mechanisms and resection barriers influencing DSB repair pathways choice. **(A)** 53BP1-dependent axis antagonizes resection and promotes ssDNA re-filling, leading to NHEJ; **(B)** DNA:RNA hybrids in the context of other barriers can be processed by alternative mechanisms or can persist at the break, unbalancing the DSB repair pathway choice. RNA molecules can be present at the break in active transcribed gene or can be newly-synthetized after a DSB. Red and light green circles indicate histone modifications. See text for details.

These and other evidence indicate that 53BP1 exerts its action as a resection barrier in an extremely dynamic way, by mutual antagonism with BRCA1 and recruiting several downstream effectors (Panier and Boulton, 2014; Zimmermann and de Lange, 2014). Notably, following DSB-induced phosphorylation by ATM, 53BP1 recruits RIF1, the Shielding complex and the CST/ Pol α-Prim complex that fills in the resected DNA end, restoring dsDNA and allowing NHEJ [see a recent review here (Setiaputra and Durocher, 2019)]. It is an open debate whether 53BP1 and its partners exert their function to limit resection directly as a physical barrier to nucleases or indirectly reconstituting processed DNA ends (Setiaputra and Durocher, 2019). Most likely, both hypotheses are true (Figure 2A).

Of note, in the S/G2 phases of the cell cycle BRCA1 promotes phosphatase PP4C-dependent 53BP1 dephosphorylation and RIF1 release (Isono et al., 2017), promoting end resection and directing repair toward HDR. Inactivation not only of 53BP1, but also of its downstream effectors was shown to increase DNA damage tolerance of cancer-prone BRCA1^−/−^ cells, most likely potentiating error prone HR pathways and increasing genome instability (Setiaputra and Durocher, 2019).

In addition to BRCA1, other factors and mechanisms modulate the mobility of the 53BP1-dependent barrier. For instance, the H2A ubiquitylation by BRCA1-BARD1 recruits the chromatin remodeler SMARCAD1, which then controls 53BP1 repositioning nearby a DSB and promotes long-range resection (Costelloe et al., 2012; Densham et al., 2016). In yeast, the SMARCAD1-ortholog Fun30 also acts on the Rad9-barrier, promoting long-range resection (Chen et al., 2012; Eapen et al., 2012; Bantele et al., 2017). Moreover, the Slx4-Rtt107 complex counteracts Rad9 binding to Dpb11/TOPBP1 and histones at the break, favoring DSB resection and HDR in yeast (Dibitetto et al., 2016).

It is important to keep in mind that the extensive resection is controlled by other factors and mechanisms than the 53BP1 barrier. For example, in human cells the 5′−3′ translocase HELB limits EXO1 and DNA2/BLM nuclease activity (Tkac et al., 2016).

## Is the DNA:RNA Hybrid a Barrier to DSB Resection?

It is an open debate how local transcription might interfere with DSB processing and repair. Indeed, DNA transcription might act as a physical barrier to DSB repair, especially during HDR, which requires long-range DSB resection. Accordingly, a reduction of DNA transcription nearby a DSB has been detected in both yeast (Lee et al., 2000; Manfrini et al., 2015) and mammals (Kruhlak et al., 2007; Chou et al., 2010; Shanbhag et al., 2010; Pankotai et al., 2012; Kakarougkas et al., 2014; Ui et al., 2015; Awwad et al., 2017; Iannelli et al., 2017; Vitor et al., 2019). While canonical ongoing transcription is switched off in response to DSB formation, mounting evidence suggests that DSB ends may act as transcriptional promoter-like elements, priming the formation of long non-coding RNA specie. In this context, transcription requires MRN-dependent recruitment of RNAPII at DNA lesions (Michelini et al., 2017) or, in the case of DSBs generated at promoter-proximal regions, cAbl-dependent tyrosine phosphorylation of RNAPII (Burger et al., 2019). The newly-synthetized non-coding RNAs at DSBs contribute to signal locally DNA damage and facilitate DNA repair (Francia et al., 2012; Wei et al., 2012) and, by changing chromatin structure, also possibly contribute to repress canonical transcription (Burger et al., 2019).

Since RNA synthesis nearby a DSB is both repressed and activated, it is unclear whether the transcription process *per se* and/or the formation of transcripts might antagonize locally the DSB resection and repair. Indeed, nascent RNA can be utilized as template to repair DSBs in transcribed genes via either error-free cNHEJ in human cells (Chakraborty et al., 2016), or HDR upon its assimilation into broken DNA by Rad52 protein, via an inverse strand exchange mechanism conserved from yeast to human (Keskin et al., 2014; Mazina et al., 2017). There are also evidence that DNA:RNA hybrids, generated at resected or minimally resected DNA ends, regulate the recruitment of RPA, BRCA1, BRCA2, RAD51, and RAD52, promoting HDR (Ohle et al., 2016; Cohen et al., 2018; D'Alessandro et al., 2018; Lu et al., 2018; Burger et al., 2019; Domingo-Prim et al., 2019). Another recent study in human cells showed that DSBs within transcriptionally active genes lead to the formation of R-loops, whose cleavage by the endonuclease XPG promotes an alternative way to initiate DSB resection and HDR (Yasuhara et al., 2018). Of interest, after their recruitment at XPG-processed DSBs, RAD52, and BRCA1 limit the 53BP1-RIF1 barrier. Remarkably, dysfunctions in the XPG-dependent mode to initiate resection lead to elevated NHEJ at transcribed loci and genome instability. Although DNA:RNA hybrids might not antagonize DSB resection initiation, they need to be dismantled by specific helicases or processed by RNases, allowing the HDR repair to proceed (Li et al., 2016; Ohle et al., 2016; Cohen et al., 2018). Interestingly, the RNase EXOSC10, a catalytic subunit of the RNA exosome complex, has been recently involved to clear DNA:RNA hybrids at DSBs, preventing hyper-resection and coupling the nucleolytic processing with deposition of RPA and HDR repair in human cells (Domingo-Prim et al., 2019). Similarly, the accumulation of hybrids in cells depleted of Senataxin, a DNA/RNA helicase with R-loop-resolving activity, counteracts the binding of RAD51 and stimulates that of 53BP1 (Cohen et al., 2018), leading to illegitimate repair of broken ends and chromosome translocations (Brustel et al., 2018; Cohen et al., 2018). In this scenario, it is also important to mention that a recent work has reported that high levels of DNA:RNA hybrids at DSBs, due to the inactivation of human RNA binding protein HNRNPD, limit DSB resection, and HDR (Alfano et al., 2019).

Overall, current findings indicate that DNA:RNA hybrids at a DSB both promote and impair resection and HDR (Figure 2B), which might depend on local chromatin context. However, timely formation and dissociation of DNA:RNA hybrids impact on the DSB repair pathway choice and genome stability. Further investigations will be required to understand how the recruitment of the RNAPII complex and RNA synthesis impact locally on DSB resection and repair, favoring NHEJ or HDR.

## Conclusive Remarks

Studies from yeast to human have shown that a wide variety of proteins and DNA/RNA transactions modulate resection, altering DSB repair pathway choice. Further investigations will be required to define their functional interplay. Moreover, an open debate regards the DSB resection initiation within active transcribed chromatin. It is unclear how the transcription machinery and the DNA:RNA hybrids influence the DSB repair pathway choice. Do the DNA:RNA hybrids at DSBs interfere with the resection antagonists, rather than with the resection machinery? Do the loading of the resection antagonists (such as KU and/or 53BP1) at the DSB is influenced by DNA:RNA hybrids?

Other relevant questions regard the role and mechanisms of resection barriers at stall or collapsed replication forks. Indeed, transcription, and the DNA damage response are highly influenced by the chromatin architecture changes occurring during DNA replication.

Remarkably, factors involved in DSB resection are deregulated in different cancers and genome instability syndromes, being also considered promising therapy targets. Indeed, the importance of all the factors involved in establishing and/or dampening resection barriers clearly emerged by treating tumor cells, which carry mutations in the BRCA1-axis, with the PARP1 inhibitor Olaparib. Notably, inactivation of the 53BP1-dependent resection barrier dramatically reduces the effectiveness of the treatment on BRCA1 defective cells, possibly leading to genome instability, poor prognosis, and cancer relapse (Lord and Ashworth, 2017; Setiaputra and Durocher, 2019).

## Author Contributions

AP conceived the idea. All authors wrote the manuscript.

### Conflict of Interest Statement

The authors declare that the research was conducted in the absence of any commercial or financial relationships that could be construed as a potential conflict of interest.
